# SPP1+ Macrophages and the Orchestration of Spatially Organized Immunosuppression in Cancer

**DOI:** 10.3390/biomedicines14020294

**Published:** 2026-01-28

**Authors:** Fanshu Li, Dafeng Xu, Zhen Tang, Yangfeng Lai, Qiumeng Liu, Huifang Liang, Hanhua Dong, Jia Song

**Affiliations:** 1 Hubei Key Laboratory of Hepato-Pancreato-Biliary Diseases, Hepatic Surgery Center, Tongji Hospital, Tongji Medical College, Huazhong University of Science and Technology, Wuhan 430030, China; lifanshu2023@126.com (F.L.); xdf0898@163.com (D.X.); lqm_910927@126.com (Q.L.); 2 Key Laboratory of Organ Transplantation, Ministry of Education, National Health Commission, Chinese Academy of Medical Sciences, Wuhan 430030, China; 3 Department of Hepatobiliary and Pancreatic Surgery, Hainan General Hospital, Haikou 570102, China; 4 Laboratory Animal Center, Tongji Hospital, Tongji Medical College, Huazhong University of Science and Technology, Wuhan 430030, China; 15927205151@163.com (Z.T.); 18064113468@163.com (Y.L.)

**Keywords:** SPP1, TAMs, TME, cancer, immunosuppression, fibrosis

## Abstract

This review describes the immunosuppressive effect of secreted phosphoprotein 1 (SPP1)+ tumor-associated macrophages (TAMs) in coordinating the tumor microenvironment (TME) as a functionally unique myeloid cell subgroup. SPP1+ TAMs transcend the traditional M1/M2 paradigm and represent a group of cells that are widely found in various cancer types. SPP1+ TAMs have the characteristics of high expression of SPP1 and promoting immune escape, matrix remodeling and metastasis. We clarify the dual developmental source of SPP1+ TAMs, and introduce the activation process of SPP1+ TAMs through recruitment, polarization and epigenetic locking. After SPP1+ TAMs are activated, they are strategically enriched in the tumor core and tumor marginal area to play their functions. Functionally, SPP1+ TAMs mainly promote the progression of tumors through three mechanisms: (1) Interacting with cancer-associated fibroblasts (CAFs): constructing an immunoexcluded fibrotic niche; (2) Multiple regulation of immune cells; (3) Promoting tumor metastasis and the construction of pre-metastatic niche (PMN). Overall, this review aims to provide a comprehensive overview of the mechanisms mediated by SPP1+ TAMs in the TME, and emphasize their unique role in cancer progression. At the same time, the treatment strategies targeting them are further explored, highlighting their potential as precise therapeutic targets for tumor treatment.

## 1. Introduction

The TME is a dynamic and complex ecosystem composed of immune cells, matrix cells, vascular systems and extracellular matrix (ECM), and its composition varies between different tumor types and different stages [1]. Macrophages in TME are called TAMs, which are one of the most important immunomodulatory cell populations in TME [2,3,4,5]. TAMs play a crucial role in tumor growth, angiogenesis, metastasis, and treatment resistance [6].

According to the traditional view, TAMs mainly come from circulating monocytes, which are guided to the tumor site through chemokines and then differentiate into tumor-promoting M2 macrophages [7,8,9]. However, the single-cell RNA sequencing (scRNA-seq) of patient samples gradually revealed that TAMs are not a homogeneous cell population, but composed of multiple functional heterogeneous subgroups [10,11,12]. Among these TAM subgroups, the high activity of the SPP1 signaling pathway suggests that it may be a key pathway regulating the interactions between TAM subpopulations and their interactions with tumor cells. Subsequent functional experiments further confirmed the central role of SPP1 in mediating these cellular interactions [13]. As a TAM subgroup with high expression of SPP1, SPP1+ TAMs have become a hot topic in current tumor immunological research because of their key role in immunosuppression, matrix remodeling and tumor metastasis [14,15].

While SPP1+ TAMs exhibit M2-like features, they are distinct from classical M2 mac-rophages. SPP1+ TAMs not only represent a TAM subgroup that promotes tumors. SPP1+ TAMs can secrete SPP1 and various cytokines and chemokines, thus interacting with various cell types [16]. SPP1+ TAMs have unique molecular markers, developmental sources and functional characteristics, which have a profound impact on the occurrence and progression of tumors [17,18,19]. Compared with previous reviews on TAM subgroups or SPP1-related research, this review has three distinct features and updated content: First, it integrates the latest single-cell and spatial omics data to systematically clarify the dual developmental origin of SPP1+ TAMs, which supplements the single-source cognition in early reviews. Second, it innovatively focuses on the spatial distribution characteristics of SPP1+ TAMs and their niche construction with CAFs, immune cells and other subsets, revealing the spatial regulatory network of SPP1+ TAMs that has not been comprehensively summarized before. Third, it systematically sorts out the multi-dimensional functional modules of SPP1+ TAMs and the underlying signal axes, and proposes precise targeted strategies based on “source-polarization-function” cascades, which goes beyond the broad-spectrum macrophage intervention ideas in previous reviews.

## 2. SPP1+ TAMs: Identification and Developmental Origins

### 2.1. Identification: How to Recognize SPP1+ TAMs

With the progress of new technologies such as scRNA-seq and spatial transcriptomics, more and more studies have found a TAM subgroup characterized by high SPP1 expression, called SPP1+ TAMs. Therefore, high SPP1 expression serves as the core molecular marker for SPP1+ TAMs [20,21]. SPP1 is a secreted glycoprotein that plays a broad role in cell adhesion, signal transduction, immune regulation, and tissue remodeling [22,23]. In the TME, SPP1 is mainly secreted by macrophages [24,25]. The expression level of SPP1 is usually strongly associated with tumor progression, immune evasion, and a poor prognosis [26]. In addition to SPP1, SPP1+ TAMs usually express a series of genes related to immunosuppression and matrix remodeling, such as APOE, APOC2, FN1, VEGFA, MMP9, GPNMB and FABP1 [27,28,29,30,31,32,33]. These molecules collectively constitute a functional barcode for SPP1+ TAMs, enabling their distinction from other macrophage subsets, particularly the pro-inflammatory CXCL9+ TAMs.

Macrophages show a high degree of heterogeneity in TME. SPP1+ TAMs and CXCL9+ TAMs represent two functional opposite polarization states: SPP1+ TAMs have immunosuppression and tumor-promoting characteristics, while CXCL9+ TAMs show immune activation and anti-tumor activity. There are significant differences in immunomodulation, metabolic characteristics and clinical outcomes between these two states, making them important biomarkers for predicting the efficacy of immunotherapy and developing intervention strategies. Unlike SPP1+ TAMs, CXCL9+ TAMs are marked by CXCL9 overexpression, and additionally highly express immune activation-related genes like CXCL10, IL-12, and MHC-II [34,35]. SPP1+ TAMs present in the tumor core are found in close proximity to CAFs. These cells inhibit the infiltration of CD8+ T cells and facilitate tumor progression. In contrast, CXCL9+ TAMs residing in the tumor periphery synergize with CD8+ T cells to form an immunostimulatory microenvironment [36,37]. In terms of clinical prognosis association, CXCL9+ TAMs and CD8+ T cells interact through chemokine and IFN-γ signaling pathways, which are typically observed in patients with favorable prognosis; whereas SPP1+ TAMs and cancer cells interact under hypoxic signaling, often found in patients with poor prognosis [38,39]. Additionally, the ratio of CXCL9+ TAMs to SPP1+ TAMs influences the efficacy of immunotherapy [40]. As a result, the ratio of CXCL9 to SPP1 is referred to as the “polarity” of TAMs, underscoring its greater practical value over traditional M1 and M2 markers, since it offers more precise and clinically significant information [15,41]. The expression ratio of CXCL9+ TAMs and SPP1+ TAMs reflects the balanced relationship between anti-tumor immune response and tumor-promoting immunosuppression in the TME. Recent research has made new progress. Researchers found in the TAMs subgroups of mice and humans that ubiquinone oxidoreductase subunit A4 (NDUFA4), as a resistor that regulates the degree of interferon stimulated genes (ISG) induction in the TAMs group, is a key determinant of the CXCL9: SPP1 TAMs ratio. Reducing NDUFA4 in macrophages can promote the accumulation of immune-stimulated anti-tumor TAMs, thus inhibiting tumor growth. This study reveals that the remodeling of the CIV subunit NDUFA4 is a key mechanism regulating macrophage adaptation to different microenvironments, holding broad implications for immunotherapy [42].Therefore, future therapeutic strategies should not be limited to broad immune activation or macrophage inhibition, but should focus on precisely modulating this balance. Shifting the therapeutic target from cellular clearance to phenotypic reprogramming and functional equilibrium restoration may offer new and effective pathways to overcome current immunotherapy resistance.

It can be seen that TAMs exhibit significant heterogeneity, a diversity not fully captured by classical models of macrophage activation [11,43]. Based on current scRNA-seq studies, in addition to SPP1+ TAMs and CXCL9+ TAMs, other frequently observed macrophage subsets include IL-1β+ TAMs, TREM2+ TAMs, FOLR2+ TAMs. Among them, The signature gene set of IL-1β+ TAMs comprises inflammation-associated molecules (IL1B, IL1A, NLRP3) and chemokines [44]. TREM2+ TAMs are enriched in lipid metabolism and immunosuppressive markers, typically ARG1, FABP5, APOE, and LDHA [45,46]. FOLR2+ TAMs are characterized by their expression of the non-canonical myeloid marker FOLR2 [47]. These gene markers facilitate the differentiation among distinct macrophage subpopulations.

In addition to identifying SPP1+ TAMs through gene markers, SPP1+ TAMs can also be identified through cell surface markers. SPP1+ TAMs typically express CD68, CD163, and CD206 [21,48]. Notably, the TAM-associated CD68 or CD206 genes are present in the SPP1+ TAM subpopulations of specific solid tumors, rather than in all solid tumor types. This finding further supports the conclusion that CD68 and CD206 serve as markers for SPP1+ TAM [49]. SPP1 functions by interacting with multiple receptors on the cell surface, the most important of which are CD44 and integrin αvβ3 [50]. Multiple studies have confirmed that signaling pathways such as SPP1/CD44 directly mediate the specific recognition and signaling communication between SPP1+ TAMs, tumor cells, and CAFs [51,52]. These molecular features collectively define the identity of SPP1+ TAMs, enabling their accurate identification and clustering in single-cell data.

### 2.2. Developmental Origin: Where Do SPP1+ TAMs Come from?

**Figure 1:**
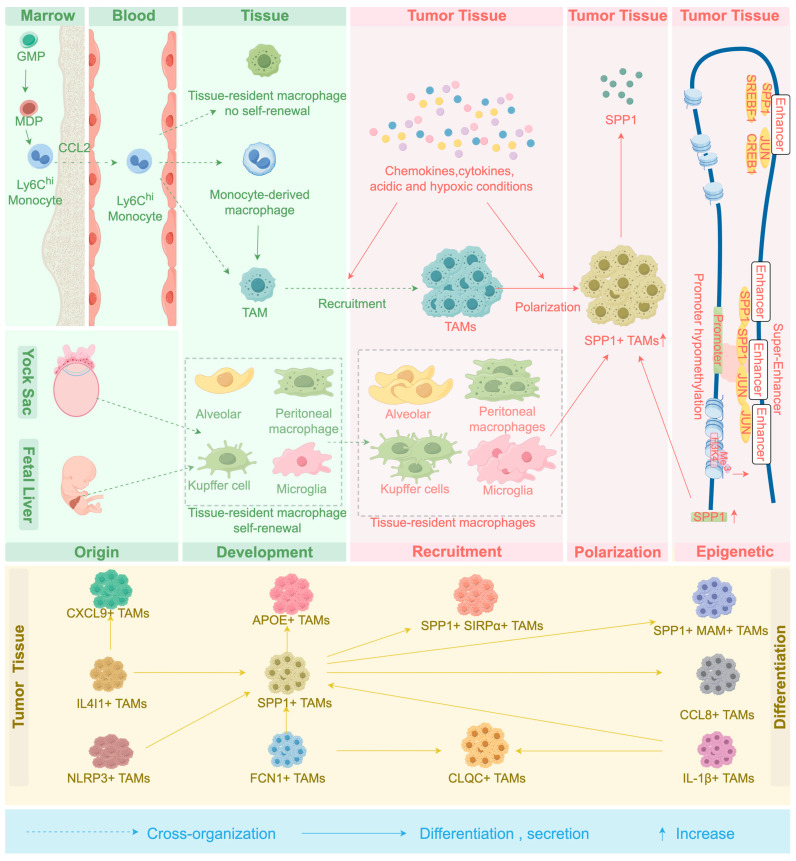
The origin, development and polarization process of SPP1+ TAMs. On the one hand, macrophages and dendritic cell predecessors (MDP) in the bone marrow produce LY6C^hi^ monocytes. LY6C^hi^ monocytes are recruited into the blood by CCL2, and then differentiate into macrophages in the tissue, and further recruited into the tumor tissue to become TAMs. Subsequently, under the action of hypoxia and acidic conditions in the TME, TAMs are polarized into SPP1+ TAMs. On the other hand, tissue-Resident Macrophages originating from the yock sac and fetal liver are also recruited in the TME, and are further polarized into SPP1+ TAMs by cytokines in the TME. Finally, under the regulation of epigenetic processes such as super enhancers, DNA methylation and histone modification, the relatively stable functional state of the SPP1+ TAMs subgroup is maintained. However, SPP1+ TAMs are not a terminal phenotype; they can serve as a transitional state and differentiate into other macrophage subsets.

Previously, it was believed that SPP1+ TAMs originate from macrophages that develop from circulating monocytes. However, recent studies have shown that SPP1+ TAMs do not solely derive from circulating monocytes but also originate from tissue-resident macrophages (Figure 1).

#### 2.2.1. Monocyte-Derived Macrophages (MDMs)

TAMs primarily originate from monocytes in the adult bone marrow. As the main reservoir for hematopoietic stem cells, the bone marrow continuously generates and releases monocytes into the peripheral blood circulation [53,54]. In the early stages of tumor development, tumor cells release large amounts of chemokines, like CCL2, as well as colony-stimulating factors, such as CSF1. These factors bind to receptors on the surface of monocytes, such as CCR2 and CSF1R, inducing the directional migration of monocytes to the tumor tissue [55,56]. The infiltrating monocytes subsequently express a variety of protumorigenic genes, including SPP1 [57].

In prostate orthotopic xenograft tumors, lineage tracing technology revealed that circulating monocytes acquire a SPP1-high phenotype within 7 days [58]. After monocytes high-express SPP1, SPP1 further promotes the recruitment of monocytes and drive macrophages to polarize towards a specific functional state [59]. As monocytes continuously infiltrate the tumor tissue, SPP1+ TAM abundance increases proportionally [60]. Multiple studies further support this view. For instance, in the TME of thyroid cancer patients, a synchronized increase in both SPP1+ TAMs and CD14+ monocyte infiltration was observed [61]. A study on colorectal cancer (CRC) also clearly indicates that SPP1+ TAMs originate from infiltrating monocytes [62]. Additionally, a scRNA-seq study of 22 treatment-naive lung adenocarcinoma patients confirmed that monocyte-derived macrophages represent an independent source of SPP1+ TAMs [19].

#### 2.2.2. Tissue-Resident Macrophages

In addition to monocytes, tissue-Resident Macrophages are also an important source of SPP1+ TAMs. Most tissue-Resident Macrophages in the human body originate from the yolk sac and fetal liver [63]. Resident macrophages derived from the yolk sac of the embryo are an important source of TAMs. In the early stage of embryonic development, the yolk sac is the main source of hematopoietic stem cells. The primitive macrophages produced by the yolk sac migrate to various tissues and gradually form tissue-Resident Macrophages [64]. Tissue-Resident Macrophages have the ability to self-renew and are mainly found in tissues such as the liver, brain and skin. A typical example is Kupffer cells [65].

Among various types of cancer, SPP1+ TAMs derived from tissue-Resident Macrophages have been identified. In hepatocellular carcinoma (HCC), the liver-resident Kupffer cells are gradually converted into SPP1+ TAMs under tumor pressure, while the expression of core transcription factors such as SPIC and MAFB decreases [66]. Another study shows that Kupffer cells directly activate and transform into SPP1+ CD206+ tumor-promoting macrophages by ingesting tumor-derived extracellular vesicles [67]. Single cell analysis also identified a large number of immunosuppressive SPP1+ VSIG4+ peritoneal-resident macrophages in peritoneal metastatic colorectal cancer (PM-CRC) [68]. Similarly, in the mouse brain disease model, it was also found that the macrophage group (small glial cells) resident in the brain expressed SPP1 [20].

These research results together show that SPP1+ TAMs have a variety of cell sources, not only from circulating monocytes, but also from tissue-Resident Macrophages. An in-depth understanding of the diverse cell sources of SPP1+ TAMs will provide an important theoretical basis for the development of precise targeted therapy, especially for the treatment of specific sources or differentiation pathways.

## 3. The Polarization Process of SPP1+ TAMs

The polarization of SPP1+ TAMs is a dynamic and complex process, including three stages: recruitment, polarization activation and epigenetic fixation.

### 3.1. The First Stage: Recruitment

In the first stage, various chemokines and cytokines secreted by tumor cells and matrix cells mediate the recruitment of monocytes to tumor tissue. Under the action of these chemokines and cytokines, monocytes are recruited to the tumor site and then differentiate into TAMs [69,70]. Among these chemokine signals, the CCL2/CCR2 signaling pathway is the critical pathway for mononuclear cell recruitment, and is one of the targets widely used in TAM research and cancer treatment [71]. The recruitment process also depends on the activation of certain signaling pathways. In response to inflammatory stimulation, the NF-κB signaling pathway regulates the expression of a series of endothelial cell genes, which helps the recruitment of monocytes [72]. The exosomes HMGB3 also regulates monocytes recruitment by activating the STAT3/MAPK/NF-κB/CCR2 signaling pathway [73]. It is worth noting that monocytes from different sources respond differently to chemokines, which may affect the distribution and differentiation fate of TAM from different sources in tumor tissue [63].

### 3.2. The Second Stage: Polarization and Differentiation

#### 3.2.1. Polarization

After monocytes are recruited into the tumor tissue, they encounter stress conditions such as hypoxia, acidic conditions and high lactate. These factors activate transcription factors such as HIF-1α, CEBPB and STAT3, causing monocytes to differentiate into TAMs and rapidly initiate the expression of SPP1. In the TME, hypoxia is one of the key factors in promoting the formation of SPP1+ TAMs. In hypoxia-exposed MDMs, the expression of SPP1 is significantly higher than normoxic MDMs. This demonstrates hypoxia can promote the polarization of macrophages to SPP1+ TAMs [74]. In a CRC liver metastasis model, the study shows that the expression of SPP1 in TAMs is upregulated through the HIF-1α/STAT3 pathway under hypoxia and acidic conditions [75]. In addition to hypoxia, lactic acid is also one of the key factors in promoting the formation of SPP1+ TAMs in TME. Clinical data analysis indicates that high expression of NAMPT in SPP1+ TAMs is associated with poor prognosis in patients with colorectal cancer. Mechanistically, in vitro experiments have confirmed that lactic acid can significantly upregulate the expression of SPP1 in macrophages in a NAMPT-dependent manner, revealing a specific pathway through which lactic acid promotes the formation of SPP1+ TAMs. [76]. In vitro experiments confirm that lactic acid treatment significantly promoted SPP1 mRNA and protein expression in macrophages. [77].

Beyond environmental factors, a variety of cytokines secreted by tumor cells and stromal cells are also involved in the activation of SPP1+ TAM polarization. One study demonstrated that elevated IL-6 and TGF-β1 signaling promote the formation of SPP1+ TAMs [78].

Furthermore, in CRC, IL-1 was found to activate CRC peripheral blood monocytes via IL-6, thereby directly promoting the differentiation of tumor-infiltrating monocytes into SPP1+ TAMs [62]. Following successful differentiation into SPP1+ TAMs, these cells secrete SPP1 to act on other TAMs subpopulations, generating TAMs subsets with multi-molecular signatures. In the lung cancer, TREM2+ TAMs can differentiate into TREM2+ SPP1+ TAMs, thus promoting tumor progression [17]. SPP1+ VSIG4+ TAMs derived from VSIG4+ TAMs have also been detected in peritoneally metastatic CRC [68] and human ATC tumors [77].

#### 3.2.2. Differentiation

In addition to polarizing directly from monocytes, SPP1+ TAMs may also differentiate from other TAM subpopulations. For example, IL-1β+ TAMs colocalize with neutrophils in moderately hypoxic and inflamed tumor regions, while SPP1+ TAMs are enriched in necrotic areas, suggesting that IL-1β+ TAMs locally evolve into SPP1+ TAMs within hypoxic tumor zones [79]. As subsets of IL4I1+ TAMs, both CXCL9+ TAMs and SPP1+ TAMs are associated with phagocytic function, but the CXCL9+ TAMs are enriched in the active tissue region and have a stronger antigen presentation function, while SPP1+ TAMs are concentrated in the hypoxic and necrotic region, which is related to neutrophil death [80]. In the global trajectory of mononuclear cells to mature TAMs differentiation, NLRP3+ TAMs are distributed between the trajectory origin and SPP1+ TAMs, which are their direct precursor subgroup [81].

In addition, SPP1+ TAMs also have transitional-state properties. In gastric cancer, it has been found that SPP1+ TAMs are a transition state between two macrophage subsets, FCN1+ TAMs and APOE+ TAMs [82]. When embryonic tissue resident macrophages differentiate into CCL18+ TAMs, they will also go through the excessive stage of SPP1+ precors. After development and maturity, CCL18+ TAMs have a stronger tumor-promoting effect than SPP1+ TAMs [49]. At the same time, there is further differentiation within SPP1+ TAMs, depicting subgroups with different functions of SPP1+ TAMs. SPP1+ TAMs further expression of SIRPα can be differentiated into SPP1+ SIRPα+ TAMs [83]. After determining the unique transcription and functional characteristics of SPP1+ TAMs, a group of subgroups of expression matrix group-related genes were identified in SPP1+ TAMs through single-cell trajectory analysis, and their functions were enriched by ECM remodeling and fine cell metabolism represents a “matrisome-associated macrophage” (MAM). Study their relationship with other TAM subgroups and their differentiation status. It is found that SPP1+ MAM+ TAMs are at the end of the trajectory. These results show that SPP1+ MAM+ TAMs may represent the conservative ultimate polarization state generated by SPP1+ TAMs [84].

Single-cell sequencing also identified a functional phenotype completely independent of SPP1+ TAMs subgroup: C1QC+ TAMs. In the global trajectory of differentiation of mononuclear cells to mature TAMs, SPP1+ TAMs and C1QC+ TAMs are at the end of different branches, respectively, indicating that the functional phenotypes and differentiation trajectories of C1QC+ TAMs and SPP1+ TAMs are completely different [81]. TAMs of mononuclear origin differentiate into mature C1QC+ TAMs via FCN1+ TAMs and pre-C1QC+ TAMs. In contrast, TAMs derived from embryonic RTMs progress through early-stage and mature SPP1+ TAMs before becoming CCL18+ TAMs. Both mature C1QC+ TAMs and SPP1+ TAMs can be further subdivided into functionally distinct subsets [49]. Most studies indicate that SPP1+ TAMs and C1QC+ TAMs represent two functionally distinct and mutually exclusive polarized states [85]. For example, among cervical cancer patients, it was found that patients with C1QC^high^ and SPP1^low^ had the best prognosis, while patients with C1QC^low^ and SPP1^high^ TAMs had the worst prognosis [86]. Anti-CD115 treatment for macrophages has been proven to prioritize reducing SPP1+ TAMs, retaining C1QC+ TAMs, and promoting tumor reversal [87]. But interestingly, although studies have found that SPP1 is mutually exclusive with C1QC in CRC and breast cancer (BRCA), SPP1 is co-expressed with C1QC in a subconcenter of uterine corpus endometrial carcinoma (UCEC) [43]. It can be seen that macrophage subgroups of different cancer types show heterogeneous transcriptome patterns, which cannot be generalized and needs to be analyzed separately to better describe their specific characteristics.

In the study of the relationship between SPP1+ TAMs and CXCL9+ TAMs, differences in molecular expression were also observed across various cancers or subtypes. The analysis of colon cancer and glioblastoma samples shows that the abundance of SPP1+ TAMs is negatively correlated with the enrichment of CXCL9+ TAMs [88,89]. However, it is worth noting that previous studies on cancers such as ovarian cancer (OC) [90] and infiltrative basal cell carcinoma (iBCC) [91] have failed to establish an exclusive expression pattern between CXCL9 and SPP1, and some have even revealed a concordant relationship. These studies further emphasize the heterogeneity of macrophage subpopulations in different types of cancer and the need for further exploration and validation in subsequent research.

### 3.3. The Third Stage: Epigenetic Locking

Epigenetic mechanisms lock the functional state of SPP1+ TAMs as tumors progress. At this stage, multiple epigenetic regulatory mechanisms work together to maintain the stable phenotype and functional state of SPP1+ TAM. Some researchers, based on GEO data and tumor immune infiltration characteristics, developed an epigenetic immune-related scoring system (EIRS) to stratify patients with poor prognosis, identifying SPP1 as an important gene in the EIRS system [92].

Super-enhancers (SEs) are large regulatory regions composed of multiple enhancer elements that can drive high-level gene expression [93,94]. The SPP1 gene locus exhibits a super-enhancer element occupied by identified transcription factors, and it has been demonstrated that SPP1 expression is super-enhancer-dependent [95]. In a clinical cohort study of HCC, a framework for identifying SEs was established, revealing 133 SPP1+ TAM-specific SEs and constructing a regulatory network composed of transcription factors, SEs, and target genes [66]. This implies that these super-enhancer elements exert a unique regulatory role in SPP1+ TAMs.

DNA methylation is a key mechanism of gene expression regulation, and abnormal methylation patterns play a central role in the onset and advancement of cancer [96]. The analysis of TCGA data reveals that hypermethylation of the SPP1 promoter is inversely related to SPP1 expression and correlates with a better prognosis in various types of cancer [97]. Conversely, hypomethylation of SPP1 is associated with its overexpression and poor prognosis in multiple cancers [22]. PRAME, which exhibits hypomethylation and upregulation in lung cancer, promotes the recruitment of monocytes to tumor tissues and facilitates their differentiation into TAMs, including SPP1+ TAMs [98]. This indicates that the hypomethylation pattern of SPP1 is also a key mechanism for maintaining the functional state of SPP1+ TAMs.

Histone modifications are also key factors regulating epigenetic states. Elevated levels of histone 3 lysine 4 tri-methylation (H3K4me3) in the SPP1 promoter correlate with increased SPP1 expression [99]. In pancreatic cancer, increased deposition of H3K4me3 at the SPP1 promoter stimulates SPP1 expression, which leads to elevated SPP1 protein levels in both tumor cells and immune cells within the TME [100]. The significantly reduced lactylation of histone 3 lysine 18 (H3K18) in TAMs lacking VSIG4 leads to decreased SPP1 transcription, collectively disrupting cell–cell interactions between TAMs and neutrophils [77]. According to the above studies, there seems to be reason to believe that histone modification also plays an important role in the regulation of SPP1+ TAMs. Although it is not clear how histone modification specifically regulates SPP1+ TAMs, some researchers believe that histone modification is one of the key factors in determining whether TAMs eventually evolve into SPP1+ TAMs or CXCL9+ TAMs [36].

After processes such as recruitment, polarization, and epigenetic locking, monocytes eventually differentiate into functionally relatively stable SPP1+ TAMs.

## 4. Spatial Distribution and Niche Construction of SPP1+ TAMs

In recent years, the application of single-cell and spatial omics has further enhanced our understanding of the spatial distribution patterns of SPP1+ TAMs in tumor tissues. This section primarily discusses the distribution patterns of SPP1+ TAMs in tumors and their co-localization patterns with other cell types based on single-cell and spatial omics data. These spatial associations strongly suggest the existence of complex cellular interactions and functional modules; however, the validation of their specific molecular mechanisms and causal relationships will require integration with the functional experimental studies described in the following sections.

### 4.1. SPP1+ TAMs in the Tumor Core

**Figure 2:**
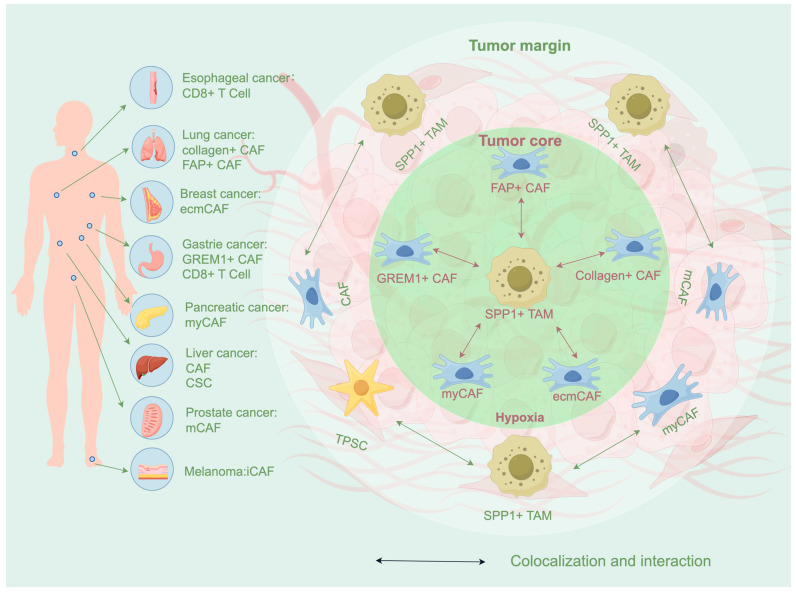
Spatial distribution and niche construction of SPP1+ TAMs in cancer. SPP1+ TAMs are distributed in the hypoxic area of the core of the tumor, and also exists at the edge of the tumor. In the tumor core, SPP1+ TAMs are co-located with a variety of subtypes of CAFs, constructing a niche of cell–matrix remodeling and fibrosis. This immunosuppressive niche can inhibit the normal immune function of CD8+ T cells, thus inhibiting tumor immunity. At the edge of the tumor, SPP1+ TAMs interact with a variety of subtypes of CAFs to restrict the infiltration of CD8+ T cells, thereby further facilitating tumor immune evasion. The spatial distribution and ecological position construction of this SPP1+ TAMs have been confirmed in a variety of cancer types.

In the tumor core, the oxygen tension is often less than 5 mmHg, so that the tumor cells in the core are in a state of hypoxia for a long time [101]. Spatial transcriptomics and multiplex immunofluorescence analysis reveal that SPP1+ TAMs are specifically enriched in the hypoxic necrotic regions of tumors, where they exhibit stable spatial co-localization with other cells in the TME. This suggests that they may collectively assemble into a functional multicellular niche [29] (Figure 2). In triple-negative breast cancer (TNBC), multiple immunofluorescence analyses verified the enrichment of SPP1+ TAMs in the hypoxic region of the tumor core [51]. With this enrichment, hypoxic signals also drive the interaction between SPP1+ TAMs and cancer cells [38]. Multiple studies have shown that SPP1+ TAMs express genes related to angiogenesis, EMT, hypoxia and immunosuppression in the TME [102,103].

In HCC, SPP1+ TAMs have been observed to colocalize with cancer stem cells (CSCs) in the hypoxic regions and are associated with the deterioration of the TME [104]. In the hypoxic region of the tumor core, SPP1+ TAMs are also co-located with fibroblasts of different subpopulations. This phenomenon is correlated with T cell exclusion in the TME, the immunosuppressive state of patients, and poor clinical outcomes. Spatial transcriptomics and multiplex immunofluorescence analyses have revealed this phenomenon in various types of cancer: in HCC, researchers have observed spatial colocalization between SPP1+ TAMs and cancer-associated fibroblasts (CAFs), a pattern linked to accelerated tumor growth [36]. In poorly differentiated lung adenocarcinoma, the coexistence of FAP+ CAF and SPP1+ TAMs is significantly correlated with poor responses to immunotherapy in patients [105]. In TNBC, SPP1+ TAMs highly express factors such as TGF-β1 and SPP1 under hypoxic conditions. These factors, in turn, induce the differentiation of normal fibroblasts into ECM-remodeling CAFs (ecmCAFs), ultimately achieving T-cell exclusion by aggravating stromal fibrosis [106]. In lung adenocarcinoma samples with TP53 mutations, SPP1+ TAMs and collagen-positive fibroblasts constitute a multicellular niche associated with a metastatic phenotype [107]. The colocalization of GREM1+ CAFs and SPP1+ TAMs in gastric cancer is associated with poor prognosis [108]. In pancreatic cancer, SPP1+ APOE+ TAMs also exhibit spatial synergy with CTHRC1+ GREM1+ myofibroblast-like CAFs (myCAFs) [48].

### 4.2. SPP1+ TAMs at the Tumor Margin

SPP1+ TAMs and fibroblasts not only colocalize in hypoxic regions and tumor cores but also colocalize at the tumor margins, forming an immunosuppressive niche. In prostate cancer, the spatial niche composed of metabolic CAFs (mCAFs) and SPP1+ TAMs is located near the tumor margins of invasive prostate cancer [109]. Similar findings were observed in PDAC, where PLXDC1+ tumor-associated pancreatic stellate cells (TPSCs) colocalize with SPP1+ TAMs and LRRC15+ myCAFs at tumor margins. Via ligands such as CCL2 and ANXA1, PLXDC1+ TPSCs engage SPP1+ TAMs, and these reciprocal interactions jointly foster a desmoplastic and immunosuppressive niche. This spatially restricted niche at the invasive front may exclude T-cell infiltration and promote the exhaustion of adjacent T cells [110]. In 8 HCC patients treated with anti–PD-1 therapy, SPP1+ TAMs around tumor cells formed a tumor immune barrier (TIB) structure with CAFs in ICB non-responders, whereas this structure was absent in responders [111]. CAF subpopulations are the most important cell populations interacting with SPP1+ TAMs. It is the colocalization of SPP1+ TAMs with the CAF subpopulations at the tumor core and tumor boundary that provides the foundation for the subsequent immunosuppressive effects of SPP1+ TAMs.

T cells are also crucial members involved in the construction of the SPP1+ TAMs niche. In the TME, it has been found that SPP1+ TAMs co-localize with proinflammatory CAFs (iCAFs) and CD8+ T cells [112]. Additionally, in esophageal squamous cell carcinoma [83] and HCC [113], co-localization of SPP1+ TAMs with T cells in fibrotic regions has been further observed, and SPP1+ TAMs appear to suppress T cells, yet the underlying molecular mechanisms remain to be fully elucidated. When CD8+ T cells become exhausted, the exhausted CD8+ T cells and SPP1+ TAMs jointly orchestrate a stromal immunosuppressive network that may create a barrier hindering antitumor immunity [114].

## 5. Core Functional Modules of SPP1+ TAMs

### 5.1. SPP1+ TAMs Interact with CAFs: Promoting Matrix Fibrosis and Immunosuppression

**Figure 3:**
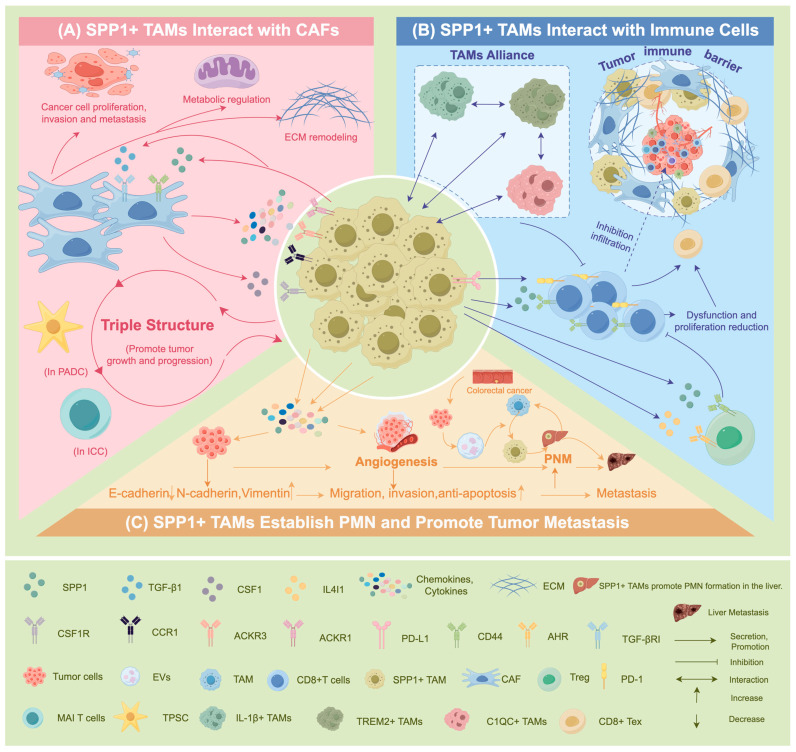
The role of SPP1+ TAMs in tumor progression. SPP1+ TAMs. SPP1+ TAMs promote tumor progression primarily through three mechanisms: (**A**) SPP1+ TAMs can activate CAFs and interact with them, promoting extracellular matrix remodeling and the formation of an immunosuppressive niche. (**B**) SPP1+ TAMs collectively suppress CD8+ T cell immune function through their alliance with other macrophage subpopulations, the immunosuppressive network formed by CAFs, and Treg-mediated regulation, which constitutes the primary pathway for their immunosuppressive effects. (**C**) SPP1+ TAMs interact with tumor cells via various chemokines and cytokines to promote EMT, neovascularization, and PMN formation, ultimately leading to tumor metastasis.

The interaction between SPP1+ TAMs and CAFs within the TME promotes cellular and matrix fibrosis and immune suppression. Together, they construct an immune-rejecting, fibrotic, hypoxic, and metastasis-promoting TME. This interaction is not unidirectional but rather a bidirectional, positive-feedback synergistic relationship. Ultimately, this impedes T-cell infiltration, renders immunotherapy ineffective, and accelerates tumor progression (Figure 3).

#### 5.1.1. Heterogeneity of CAFs: The Interaction Partners of SPP1+ TAMs Are Not Random

CAFs exhibit marked heterogeneity and are not a homogeneous cell population [115,116]. CAFs can be primarily classified into three subpopulations: iCAFs, myCAFs, and antigen-presenting CAFs (apCAFs) [117]. As the previous section, we have described various CAFs colocalizing with SPP1+ TAMs within the TME, with myCAFs exhibiting the closest interaction with SPP1+ TAMs. myCAF and its differentiated form ecmCAF are the primary cells responsible for producing and contracting collagen fibers, which can lead to tissue fibrosis [118]. In TNBC, SPP1+ TAMs promote the differentiation of normal fibroblasts into ecmCAFs under hypoxic conditions [106]. Additionally, iCAFs can secrete CCL2 to engage CCR1 on SPP1+ TAMs, leading to activation of NFKBIA (Nuclear Factor kappa B Inhibitor Alpha) in SPP1+ TAMs and subsequent up-regulation of IL-6, which may contribute to the exhaustion of CD8+ T cells [112]. Notably, one study indicates that iCAFs are associated with a better prognosis in cancer patients [119]. apCAFs have also been found to regulate SPP1. Not only apCAFs upregulate SPP1 expression [120], but they also interact with non-small cell lung cancer (NSCLC) cells via the SPP1-CD44/SPP1-PTGER4 axis, thereby promoting NSCLC metastasis [121]. The heterogeneity of CAFs enables them to interact with SPP1+ TAMs through distinct subtypes within the TME, thereby facilitating the multifaceted functions of CAFs (Table 1).

#### 5.1.2. Signal Axis: Mediating the Interaction Between SPP1+ TAMs and CAFs

The abundant SPP1 secreted by SPP1+ TAMs is the core mediator of their interaction with CAFs. CD44 is the key site that mediates this interaction. The absence of SPP1-binding capacity in certain CD44 isoforms implies greater complexity in SPP1+ TAMs interactions than is currently understood [134]. SPP1 preferentially binds to the alternatively spliced CD44v isoforms within the CD44 family [135]. Among them, SPP1 primarily regulates cell motility, invasion, chemotaxis, and survival by binding to the CD44 v3-v6 splice variant [136].In the liver, the high expression of SPP1 preferentially targets the CD44 on adjacent HSCs, thereby activating the PI3K/AKT signaling pathway and promoting the differentiation of HSCs into CAFs [137]. In lung adenocarcinoma, the interaction between ecmCAFs and SPP1+ TAMs through COL1A1/CD44 and COL1A2/CD44 ligand-receptor pairs is an important mechanism for the formation of an immune rejection microenvironment [126].

Similar to the activation of CAFs through CD44, the enrichment induced by SPP1 of TAMs further promotes the secretion of TGF-β1 by TAMs, thus activating CAFs and promoting tumor fibrosis [138]. In hypopharyngeal squamous cell carcinoma (HPSCC) [81] and TNBC [106] SPP1+ TAMs have been found to promote the activation of CAFs. by secreting TGF-β1 signals.

In melanoma [139] and CRC [140], elevated expression of CSF1R in SPP1+ TAMs has been found, and CSF1R responds to CSF1 secreted by CAFs. After CSF1 is secreted by CAFs, it binds to the CSF1R on the surface of SPP1+ TAMs, promoting the survival and proliferation of SPP1+ TAMs. In turn, SPP1+ TAMs release cytokines like CXCL ligands, which then interact with FAP+ CAFs, thereby further modulating the TME [130]. Interestingly, a CRC-based study found that the expression of CSF1R in C1QC+ TAMs is more significant than in the concentration of SPP1+ TAMs [102]. Another study indicated that in mice and humans, treatment with anti-CSF1R monotherapy preferentially depletes C1QC+ TAMs with inflammatory characteristics, while having no effect on SPP1+ TAMs that express pro-angiogenic and tumorigenic genes [141]. Further research points out that the above phenomenon is due to the priority expression of genes involved in angiogenesis in anti-CSF1R-resistant TAMs [88]. As mentioned earlier, SPP1+ TAMs exhibit pro-tumor characteristics, while C1QC+ TAMs demonstrate anti-tumor properties. Therefore, the use of anti-CSF1R monotherapy may have poor efficacy in cancer patients.

In addition to the above signaling pathways, other signaling pathways have been found to mediate the interaction between SPP1+ TAMs and CAFs. The IL-6/STAT3 pathway can promote the interaction between POSTN+ CAFs and SPP1+ TAMs [123]. iCAFs can secrete CCL2, which binds to CCR1 on SPP1+ TAMs, thus activating the NF-κB pathway [112].

#### 5.1.3. Formation of the “Multi-Linked” Immunosuppressive Alliance

SPP1+ TAMs exhibit lipid metabolism abnormalities and extracellular matrix remodeling activity [142], and also express genes related to extracellular matrix remodeling, tumor invasion, and immune suppression [124,143]. Therefore, when SPP1+ TAMs interact with CAFs, they promote connective tissue proliferation, extracellular matrix remodeling, and angiogenesis [103,144,145], further establishing an immunosuppressive alliance [109,125]. Beyond SPP1+ TAMs and CAFs contributing to the immunosuppressive alliance, other cells within the TME also colocalize with SPP1+ TAMs and CAFs, forming a multi-component immunosuppressive alliance. For example, in pancreatic ductal adenocarcinoma (PDAC), the immunosuppressive alliance composed of PLXDC1+ TPSCs, LRRC15+ myCAFs, and SPP1+ TAMs may exclude T-cell infiltration and promote T-cell exhaustion [110]. In intrahepatic cholangiocarcinoma (ICC), POSTN+ FAP+ fibroblasts, SPP1+ TAMs, and MAIT cells form a “tripartite structure” that promotes tumor growth and ICC progression [132]. This unique spatial organization pattern suggests that these “tripartite structure” may physically construct and functionally sustain an immune-excluded microenvironment within the tumor. Additionally, under hypoxic microenvironmental conditions, SPP1 expression increases within tumors. SPP1+ TAMs interact with CAFs to promote the reshaping of the extracellular matrix and the formation of TIB, thus limiting the infiltration of immune cells [111]. In summary, the interaction between SPP1+ TAMs and CAFs, as well as their interconnection with other microenvironmental cells, forms a three-dimensional immune rejection barrier, limiting the infiltration of immune cells and promoting tumor growth. Targeting SPP1+ TAMs can reverse immune rejection and enhance the response to immunotherapy [106].

In summary, spatial omics has revealed that SPP1+ TAMs and CAFs form a stable immunosuppressive alliance and spatially co-localized network within tumors. Mechanistic studies further demonstrate that the two populations engage in bidirectional crosstalk via the SPP1/CD44, TGF-β1/TGF-βR1 and CSF1/CSF1R signaling axes. These observational and experimental findings converge on a model in which the synergistic interplay between SPP1+ TAMs and CAFs acts as a key driver of immune exclusion and the establishment of a fibrotic niche, ultimately blocking T cell infiltration and fostering resistance to immunotherapy. This also represents the first step in the immunosuppressive program executed by SPP1+ TAMs.

### 5.2. SPP1+ TAMs Interact with Immune Cells: Regulating the Immune Microenvironment

The effectiveness of tumor immunotherapy is closely linked to both the extent of immune cell infiltration and their functional activity in the TME. SPP1+ TAMs are key participants in the tumor immunosuppressive microenvironment. SPP1+ TAMs interact with immune cells in multiple mechanisms, such as inducing T cell exhaustion, expanding regulatory immune cells, and hindering antigen presentation, thus constructing a highly immunosuppressive TME.

#### 5.2.1. Inhibiting of T Cell Reaction

The immunosuppressive alliance created through the interaction between SPP1+ TAMs and CAFs can limit the infiltration of CD8+ T cells. This is the first step for SPP1+ TAMs to limit the immune effect of CD8+ T cells. Beyond limiting the infiltration of CD8+ T cells, SPP1+ TAMs also inhibit the immune effect of CD8+ T cells in a variety of ways. SPP1 secreted by SPP1+ TAMs inhibits the proliferation of CD8+ T cells and further reduce the number of CD8+ T cells in the tumor region [146]. SPP1+ TAMs can also induce functional damage to CD8+ T cells and inhibit their ability to perform normal cell functions. This leads to the deficiency of functional CD8+ T cells in the TME, which further promotes the immune escape of the tumor [28,58,147,148]. This makes CD8+ T cells in the TME exhausted.

The SPP1 secreted by SPP1+ TAMs can induce CD8+ T cell exhaustion via the CD44 axis, thereby suppressing anti-tumor immunity. Blocking either SPP1 or CD44 can restore CD8+ T cell function and inhibit tumor growth [149]. SPP1+ TAMs induce CD8+ T cell exhaustion and subsequently interact with the exhausted CD8+ T cells to form a stromal immune-suppressive network, creating a barrier that hinders anti-tumor immunity [114,150]. Thus, a vicious cycle is formed: SPP1 interacts with CAFs to restrict CD8+ T cell infiltration and promote CD8+ T cell exhaustion. Exhausted CD8+ T cells form an immunosuppressive network, further exacerbating the limitation on CD8+ T cell infiltration.

#### 5.2.2. Recruitment of Tregs

SPP1+ TAMs also actively recruit various regulatory immune cells. The interaction between SPP1+ TAMs and regulatory T cells (Tregs) further promotes the formation of the immunosuppressive microenvironment. In multifocal bladder cancer (BLCA), SPP1+ TAMs within recurrent tumors highly express IL4I1. IL4I1can activate the aryl hydrocarbon receptor (AHR) signaling pathway, recruiting Tregs. This, in turn, suppresses anti-tumor immunity and drives BLCA recurrence [151]. In CRC and its liver metastases, a significant accumulation of SPP1+ TAMs and Tregs has also been observed [152,153]. Via the SPP1-CD44 pathway, SPP1+ TAMs recruit Tregs into tumor tissues and induce CD8+ T cell exhaustion, ultimately contributing to an immunotherapy-resistant microenvironment [154]. The results of scRNA-seq further confirmed that this immunosuppressive microenvironment rich in SPP1+ TAMs, Tregs, and exhausted CD8+ T cells is a common feature of different cancer types [143,155,156].

#### 5.2.3. Collaborative Myeloid Cell Alliances and Metabolic Interference

SPP1+ TAMs coordinate and cooperate with the entire myeloid cell spectrum to establish multi-dimensional immunosuppressive defense. SPP1+ TAMs not only induce the activation of myeloid-derived suppressor cells (MDSCs) and enhance their immunosuppressive function [157], but also impair the maturation process of dendritic cells (DCs), inhibit their antigen presentation ability, and eventually lead to a decrease in tumor immunogenicity [13,158]. More importantly, SPP1+ TAMs coordinate with other immunosuppressive macrophage subpopulations, such as TREM2+ TAMs and C1QC+ TAMs, to form a matrix immunosuppressive network. Together, they appear to promote the exhaustion of T cells and mediate resistance to immunotherapy by sharing signaling pathways, including MIF-CD74/CXCR4 [114,159]. In addition, SPP1+ TAMs exhibit high glycolysis and lipid metabolism characteristics, creating a microenvironment for local nutritional deficiency and forming a “metabolic alliance” with other metabolically abnormal cells in TME. This “metabolic alliance” directly deprives T cells of the nutrition they need from the perspective of energy supply and inhibits anti-tumor immunity [160,161].

### 5.3. SPP1+ TAMs Establish PMN and Promote Tumor Metastasis

Tumor metastasis is a complex biological process. Its successful transmission depends not only on the characteristics of tumor cells themselves, but also on the active reshaping of the microenvironment of distant organs by primary tumors through circulating signals. This pre-modified microenvironment is referred to as the PMN [162]. Recent single-cell and spatial transcriptomics studies have revealed that SPP1+ TAMs promote tumor metastasis and the construction of PMN through multiple pathways. Research even indicates that SPP1+ TAMs are absent in non-metastatic HCC but are highly enriched in liver metastases, supporting the notion that SPP1+ TAMs are key drivers of tumor metastasis [30].

#### 5.3.1. EMT and Angiogenesis: Synergistic Mechanisms in Tumor Metastasis

SPP1+ TAMs, along with various CAFs and CD8+ T cells, form an immunosuppressive niche in the TME. This cell alliance is enriched in pathways related to hypoxia, angiogenesis, and EMT, which are closely associated with tumor metastasis. [102,103,163].

Hypoxia and acidic conditions in the TME act as upstream initiating factors, upregulating the expression and secretion of SPP1 in TAMs through the HIF-1α/STAT3 signaling pathway [75]. As a key driver of EMT, the secreted SPP1 binds to CD44 or integrin receptors on the surface of tumor cells and activates two core downstream signaling pathways: PI3K/AKT pathway and MAPK pathway [164,165]. The activation of these signaling pathways eventually leads to the down-regulation of epithelial markers and the up-regulation of interstitial markers [166,167]. SPP1+ TAMs also significantly increase vascular permeability through the HIF1α/SPP1 signaling axis, thereby creating a pathway for tumor metastasis [160]. These processes enable tumor cells to acquire stronger migration, invasion, and anti-apoptotic capabilities, laying the foundation for their escape from the primary tumor site into the blood circulation.

SPP1+ TAMs are often described as a subpopulation of TAMs that promote angiogenesis [168,169,170,171]. SPP1+ TAMs activate endothelial cells and promote the formation of new blood vessels by secreting key factors such as SPP1 and VEGFA [16]. In ovarian cancer [160], bladder cancer [172], and pancreatic neuroendocrine tumors [173], SPP1+ TAMs have been confirmed to promote tumor-associated angiogenesis through the HIF1α/SPP axis, the IGF2BP2/CCL2 axis, and the miR-183-5p/PDCD4/PI3Kγ axis. SPP1+ TAMs also degrade extracellular matrix by upregulating enzymes such as MMP9, providing a channel for vascular invasion [174].

#### 5.3.2. Construction of PMNs: Creating Prerequisites for Tumor Metastasis

The establishment of PMN requires the combined action of multiple cytokines [162]. In head and neck squamous cell carcinoma (HNSCC), SPP1+ TAMs promote vascular extravasation of tumor cells by secreting SPP1, CCL18 and CXCL8, further leading to intratumor infiltration and metastasis [171]. SPP1+ TAMs also activate CAFs through CD44. Once activated, CAFs enhance the expression of CXCL12 and promote the migration of tumor cells [52]. In CRC, studies have also shown that SPP1+ TAMs promote the invasive capacity of aggressive cancer cells by secreting CCL2 and CXCL10 [18]. In addition, SPP1+ TAMs can also enhance glycolysis [175] and reprogram lipid metabolism [176], thus affecting the TME and promoting tumor proliferation and metastasis. The interaction of SPP1+ TAMs and CAFs is also a key step in the formation of PMN. In CRC liver metastasis, SPP1+ TAMs promote the release of extracellular vesicles (EVs) of CAFs through the APOE/LRP1 axis. These EVs can transfer tumor-promoting factors and promote tumor metastasis in CRC liver metastasis [144]. Tumor-derived extracellular vesicles (TEVs) rich in circ-0034880 can be ingested by liver macrophages, thus inducing liver macrophages to activate SPP1+ TAMs. These SPP1+ TAMs further promote the formation of PMN in CRC liver metastasis [67].

## 6. Tumor Therapeutic Strategies: Targeting SPP1+ TAMs

**Figure 4:**
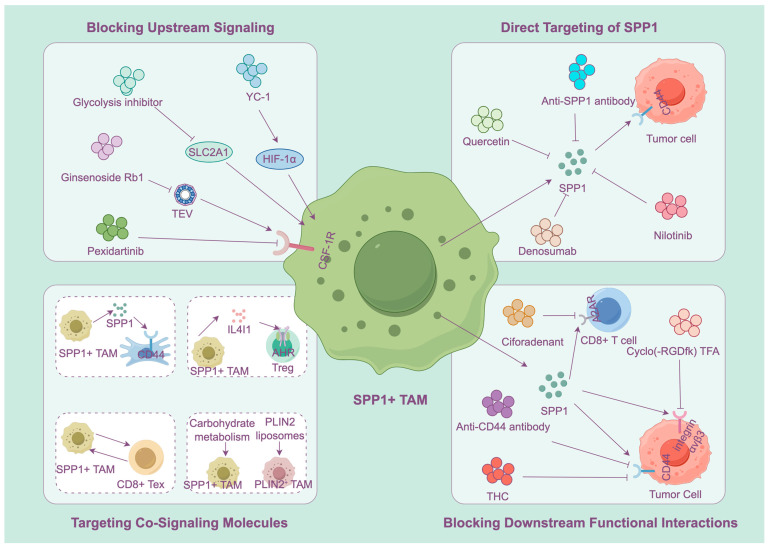
Targeted therapeutic strategies for SPP1+ TAMs. SPP1+ TAMs represent a potential novel target in cancer therapy, with treatment strategies aimed at targeting SPP1 itself, upstream signals of SPP1+ TAMs, or disrupting interactions between SPP1+ TAMs and other cells, as well as co-targeting cellular alliances that interact with SPP1+ TAMs. However, these antibodies and drugs are currently still in the experimental stage, and their safety and efficacy in humans have not yet been established. Further research is still needed to validate their safety and effectiveness and to develop new therapeutic strategies targeting SPP1+ TAMs.

SPP1+ TAMs are a key hub for shaping an immunosuppressive microenvironment, promoting drug resistance and tumor metastasis. The treatment strategy targeting this cell population has formed a comprehensive system, covering a variety of methods from direct targeting to comprehensive intervention (Figure 4) (Table 2).

### 6.1. Direct Targeting of SPP1

The purpose of targeting SPP1 itself is to directly neutralize the biological function of SPP1. Anti-SPP1 antibodies have demonstrated significant efficacy in many preclinical cancer models. Preclinical studies demonstrated that inhibition of SPP1 signaling or macrophage-specific Spp1 knockout disrupts the TIB architecture and enhances the immunotherapy sensitivity of HCC and produce synergistic anti-tumor effects with anti-PD-1 treatment [111]. Using an anti-SPP1 antibody to inhibit SPP1 signaling eliminates immunosuppressive effects in the TME and markedly extends survival in IFN signaling-deficient and CAR T cell therapy-resistant glioblastoma (GBM) mouse models. This shows that SPP1 is a common target to enhance the efficacy of CAR T cells [177]. In gastric cancer models, the SPP1/CD44 signaling axis and the MIF-CD74/CXCR4/CD44 pathway jointly form the core of an immunosuppressive barrier. This network directly drives the terminal exhaustion transition from CD8_Tex_C1 to CD8_Tex_C2 cells. (CD8_Tex_C2, as the most downstream and profoundly exhausted cell population, is regulated by Tregs and CD8_Tex_C1 cells.) Anti-SPP1 antibodies can inhibit these signaling pathways and reverse T-cell exhaustion [114]. In terms of small molecule inhibitors, nilotinib has been confirmed to bind to SPP1 with high affinity, blocking the activation of its downstream signaling, thereby reversing SPP1-mediated T cell exhaustion and immunosuppression [149]. In addition, bisphosphonates have been proven to down-regulate tumor SPP1 expression and activate T cells, revealing its potential in the treatment of solid tumors [178,179]. Natural products and traditional Chinese medicine ingredients can also regulate SPP1+ TAMs. For example, tetrahydrocurcumin (THC) reduces the infiltration of SPP1+ TAMs by inhibiting the SPP1/CD44 axis, reshapes the immune microenvironment, and shows therapeutic potential in CRC models [180]. The active ingredient of traditional Chinese medicine quercetin, has been proven to be able to down-regulate the expression of SPP1. By targeting SPP1, it enhances the efficacy of T cell immunotherapy and provides a new option for immunotherapy of thyroid cancer [181].

### 6.2. Blocking Upstream Signaling: Modulating SPP1 Expression and SPP1+ TAM Polarization

The focus of this method is to inhibit the recruitment and polarization of SPP1+ TAMs. Hypoxia and acidic conditions in the TME are the key upstream signals that drive SPP1 expression. These upstream signals upregulate the expression of SPP1 in TAMs through the HIF-1α/STAT3 pathway. Inhibiting HIF-1α can effectively reduce SPP1 levels and inhibit tumor metastasis [75]. In addition, glycolysis inhibitors can indirectly inhibit the polarization of SPP1+ TAMs by alleviating hypoxia [182].

Targeting key recruitment and polarization pathways is also an important way to prevent the recruitment and activation of SPP1+ TAMs. The CCL2/CCR2 axis is the core pathway for macrophage recruitment. In bladder cancer, the m6A reader protein IGF2BP2 mediates recruitment of SPP1+ TAMs by upregulating CCL2. Targeting IGF2BP2 can prevent this recruitment process in bladder cancer, thus reducing the infiltration of SPP1+ TAMs [172]. The complement C3/C3aR1 axis has been identified as a key driving factor for the recruitment and polarization of SPP1+ TAMs in peritoneal metastasis of gastric cancer. Targeting the axis can significantly enhance the effect of immunotherapy in peritoneal metastasis of gastric cancer [127]. The CSF1/CSF1R axis is also a key signal for regulating macrophage survival and polarization. In prostate cancer, it drives the expression of SPP1, indicating that CSF1R inhibitors may be synergistic with SPP1 targeted therapy [183]. In pancreatic neuroendocrine tumors, tumor-derived exosomes induce SPP1 expression and M2 polarization of macrophages through miR-183-5p/PDCD4/PI3Kγ/AKT/mTOR signal cascading response. Multiple nodes in this pathway are potential targets to suppress the polarization of SPP1+ TAMs [173]. Ginsenoside Rb1 can inhibit the circ-0034880 in tumor-derived exosomes, thus blocking the polarization of SPP1+ TAMs [67]. It is clear that numerous upstream signaling pathways play a role in regulating the polarization of SPP1+ TAMs. By targeting these signaling pathways, the infiltration and polarization of SPP1+ TAMs in tumor tissue can be effectively reduced, providing a new perspective for the targeting strategy of SPP1+ TAMs.

### 6.3. Blocking Downstream Functional Interactions: Suppressing the Function of SPP1+ TAM

This approach aims to disrupt the interaction between SPP1+ TAMs and receptors on the surface of other cells in the TME, thereby weakening their tumor-promoting functions. SPP1 primarily activates downstream pro-cancer signaling by binding to its core receptors, CD44 and integrins. Studies in ovarian cancer [149], HCC, [92,104], and HNSCC [171] have confirmed that disrupting the SPP1-CD44 axis can restore the function of CD8+ T cells and suppress tumor progression. Blocking the SPP1-αvβ3 integrin axis has been confirmed to effectively inhibit EMT and CRC liver metastasis [75]. In addition to the two main signaling axes mentioned above, SPP1+ TAMs can also influence downstream molecular interactions through multiple signaling axes. Targeting the SPP1-ITGA5 axis can enhance the efficacy of anti-PD-1 therapy in glioblastoma [184]. In pituitary neuroendocrine tumors (PitNETs), SPP1+ TAMs promote tumor invasion through the SPP1-ITGAV/ITGB1 axis. This suggests this axis is an important target for therapeutic intervention [185]. SPP1+ TAMs can also mediate immune suppression via the adenosine signaling pathway. Targeting the adenosine A2A receptor (A2AR) can reduce the number of SPP1+ TAMs and enhance the efficacy of anti-PD-1 therapy [58].

### 6.4. Targeting Co-Signaling Molecules

SPP1+ TAMs do not exist in isolation. Their synergy with other cells in the tumor forms the basis of the immunosuppressive microenvironment. Targeting the interaction between these cells is crucial to overcoming therapeutic drug resistance. The study found that SPP1+ TAMs are spatially co-located and functionally cooperative with CAFs in a variety of cancer types, such as CRC, pancreatic cancer, lung cancer and NSCLC. They jointly promote immune escape through the interaction of various signaling molecules (such as SPP1, VEGF, MIF and TGF-β). Therefore, combined targeting of SPP1+ TAMs and CAFs represents a very promising strategy. Combined inhibition of CAFs and SPP1+ TAMs, or blocking the COL1A1-CD44 interaction axis between them, can effectively destroy the immunosuppressive microenvironment [126].

SPP1+ TAMs form functional units with Tregs and exhausted CD8+ T cells by secreting chemical chemokines such as CCL18 and CXCL8. Targeting these chemokines or their receptors can break the aggregation of these immunosuppressive cells [151]. SPP1+ TAMs are usually in a state of high glycolysis and lipid metabolism. In the ascites of ovarian cancer, PLIN2+ lipid-related macrophages promote tumor progression through the HIF1α/SPP1 signaling pathway. Targeted PLIN2 liposomes can effectively inhibit the formation of ascites and tumor metastasis [160]. In HNSCC, SPP1+ TAMs show high fructose metabolism, and inhibiting this metabolism can reshape the immune microenvironment [14]. Therefore, the combination of metabolic inhibitors with immunotherapy is a strategy to regulate the functional state of SPP1+ TAMs and enhance the effect of immunotherapy.

Although targeted treatment for SPP1+ TAMs is still in the early stage, there is a clear targeted system for SPP1+ TAMs. In terms of tumor biomarkers, serum SPP1 levels [186] or intratumoral SPP1+ TAMs abundance [187,188] serve as precise prognostic markers to predict outcomes and identify patients most likely to benefit from therapy. The core direction in the future will focus on promoting the clinical transformation of drugs directly targeting SPP1, and exploring the optimal combination strategies with existing treatment plans. In addition, the development of bispecific antibodies or combined therapies with targeted “SPP1+ TAMs-CAFs” will be the key to completely dismantling the basis of immunosuppressive microenvironmental structures.

## 7. Conclusions and Future Perspectives

SPP1+ TAMs represent a key myeloid cell subgroup. Their functional complexity goes beyond the classic M1/M2 polarization paradigm and redefine the regulatory mechanism in the TME. Their dual developmental sources, spatial distribution characteristics and spatial interactions with CAFs and immune cells highlight their microenvironmental role as immunosuppressive and pro-metastatic.

Although clinical studies have confirmed that SPP1+ TAMs are potential therapeutic targets, there are still some key questions that have not been answered: how do holistic factors, such as aging and metabolic diseases, regulate their abundance and functional status? Can SPP1+ TAMs be reprogrammed into an anti-tumor phenotype similar to CXCL9+ TAMs, not just for eliminating them? How to optimize combination therapy with SPP1 inhibitors, anti-PD-1, and CAF-targeted drugs to disrupt the multicellular immunosuppression network?

As research continues to reveal the plasticity and environmental specificity functions of SPP1+ TAMs, combining spatial multi-omics, lineage tracking and clinical data will be the key to unlocking their therapeutic potential. Precise targeting of these core drivers is not only expected to reshape the TME and enhance the effectiveness of immunotherapy, but also open up innovative ways for immune combination therapies and precision oncology.

## Figures and Tables

**Table 1:** The role of interactions between SPP1+ TAMs and multiple CAF subtypes across different cancers.

Tumor Type	CAF Subtypes	Primary Functions and Impacts	Reference
Colorectal cancer	SPP1+ TAMs ↔ FAP+ CAFs	Forming a structured network promotes fibrosis, restricts T-cell infiltration, and leads to resistance to immunotherapy.	[21]
	SPP1+ TAMs ↔ ANGPTL2+ CAFs	As a metastasis accelerator, it jointly promotes liver metastasis.	[122]
Triple-negative breast cancer	SPP1+ TAMs ↔ ecmCAFs	Under hypoxic conditions, SPP1+ TAMs promote the differentiation of normal mammary fibroblasts into ecmCAFs, thereby enhancing ECM remodeling and stromal fibrosis.	[106]
Liver cancer	SPP1+ TAMs ↔ POSTN+ CAFs	High infiltration of POSTN+ CAFs and SPP1+ TAMs is associated with immunotherapy resistance.	[123]
Non-small cell lung cancer	SPP1+ TAMs ↔ POSTN+ CAFs	Co-localization is associated with T cell exhaustion and low infiltration, and is linked to poor prognosis.	[124]
	SPP1+ TAMs ↔ FAP+ CAFs	Patients with high expression of SPP1+ TAMs and FAP+ CAFs in the tumor show significantly poor outcomes during immunotherapy.	[125]
	SPP1+ TAMs ↔ ecmCAFs	Through the interaction of the SPP1-CD44 axis, an immune-rejecting microenvironment is formed.	[126]
Gastric cancer	SPP1+ TAMs ↔ GREM1+ CAFs	Patients with significant co-localization of SPP1+ TAMs and GREM1+ CAFs exhibit markedly shortened overall survival (OS).	[108]
	SPP1+ TAMs ↔ THBS2+ mCAFs	The formation of an immunosuppressive microenvironment in peritoneal metastasis mediates resistance to immunotherapy.	[127]
Pancreatic cancer	SPP1+ TAMs ↔ myCAFs	Associated with poor prognosis.	[15]
	SPP1+ TAMs ↔ CTHRC1+ CAFs	Through active ECM deposition and EMT, leading to worse prognosis.	[48]
	SPP1+ TAMs ↔ POSTN+ CAFs	Targeting the stromal interactions mediated by POSTN+ CAFs and SPP1+ TAMs may offer a novel therapeutic strategy for PDAC.	[128]
Bladder cancer	SPP1+ TAMs ↔ ACSL4+ CAFs	Interactions promote tumor progression and resistance to immunotherapy.	[129]
Prostate cancer	SPP1+ TAMs ↔ mCAFs	Co-localization of SPP1+ TAMs and mCAFs at the tumor margin is associated with poor patient prognosis.	[109]
	SPP1+ TAMs ↔ FAP+ CAFs	Through signaling interactions involving CSF1/CSF1R and other molecules, an immunosuppressive microenvironment is established, which correlates with poor prognosis.	[130]
Head and neck squamous cell carcinoma	SPP1+ TAMs ↔ POSTN+ CAFs	As tumors progress and infiltrate, they shape a fibrotic microenvironment that promotes tumor progression.	[131]
Intrahepatic cholangiocarcinoma	SPP1+ TAMs ↔ POSTN+ FAP+ CAFs	Forming a “tripartite structure” with MAIT cells, promoting CD8+ T cell exhaustion and tumor progression.	[132]
Lung cancer in Idiopathic Pulmonary Fibrosis	SPP1+ TAMs ↔ iCAFs	Promotes the progression of Idiopathic Pulmonary Fibrosis toward high-risk subtypes of lung cancer.	[133]

**Table 2:** Core functions and mechanisms of SPP1+ TAMs, along with therapeutic strategies and drugs targeting them.

Core Function	Key Mechanisms	Therapeutic Strategies	Representative Drugs
Promoting stromal fibrosis and immunosuppression	TGF-β1/TGF-βR1 signaling axis activates FAP+ CAFs and ecmCAFs [81]; SPP1 binds to CAF subtypes mediated by CD44, activating the PI3K/AKT pathway [126,137]; Formation of “tripartite structure” or other multi-component immune barriers with CAFs and other cells [132]; CSF1/CSF1R pathway regulates bidirectional crosstalk between SPP1+ TAMs and CAFs [139];	Blocking the interaction between SPP1 and CAFs; Inhibiting CAF activation pathways; Combined targeting of SPP1+ TAMs and CAFs Blocking the interaction between SPP1 and CAFs; Inhibiting CAF activation pathways; Combined targeting of SPP1+ TAMs and CAFs	Anti-SPP1 antibody; Tetrahydrocurcumin (THC); CSF1R inhibitors (e.g., Pexidartinib); Anti-CD44 antibody; TGF-β inhibitors
Regulating the immune microenvironment	Synergizing with other immunosuppressive macrophage subsets (e.g., TREM2+ TAMs, C1QC+ TAMs) to inhibit dendritic cell (DC) maturation [114,159]; SPP1 secreted by SPP1+ TAMs induces CD8+ T cell exhaustion via the CD44 axis [149]; High expression of IL4I1 activates the aryl hydrocarbon receptor (AHR) signaling pathway to recruit regulatory T cells (Tregs) [151]; Forming a “metabolic alliance” with other metabolically abnormal cells to deplete nutrients required by T cells [160,161]	Blocking SPP1-mediated CD8+ T cell exhaustion; Targeting the recruitment of regulatory immune cells; Combining metabolic inhibitors with immunotherapy	Nilotinib; Quercetin; A2AR inhibitors (e.g., Ciforadenant); PD-1 inhibitors; PLIN2 liposomes; Glycolysis inhibitors (targeting SLC2A1)
Promoting tumor metastasis	Secreting chemokines and activating CAFs via CD44, thereby constructing a pre-metastatic niche (PMN) [67] Hypoxia/acidic conditions in the tumor microenvironment activate the HIF-1α/STAT3 signaling pathway to upregulate SPP1 expression, thereby inducing epithelial–mesenchymal transition (EMT) and activating the PI3K/AKT and MAPK pathways [164,165]; Secreting SPP1 and VEGFA to activate endothelial cells and promote angiogenesis, and upregulating enzymes such as MMP9 to degrade the extracellular matrix (ECM) [174];	Inhibiting EMT-related signaling pathways; Blocking angiogenesis; Disrupting PMN construction; Inhibiting EV-mediated polarization	HIF-1α inhibitors (e.g., YC-1); Integrin antagonists (e.g., Cyclo(-RGDfk) TFA); VEGFA inhibitors; Ginsenoside Rb1 (inhibiting circ-0034880)
Polarization and recruitment	Hypoxia/lactic acid activates transcription factors such as HIF-1α, CEBPB, and STAT3 to initiate SPP1+ TAM polarization [74,76]; Epigenetic locking (super-enhancers, DNA hypomethylation, H3K4me3 modification) maintains the stable phenotype of SPP1+ TAMs [100] The complement C3/C3aR1 axis regulates SPP1+ TAM polarization [127]; The CCL2/CCR2 axis mediates monocyte recruitment to the tumor site [172];	Blocking monocyte recruitment; Inhibiting upstream signals of polarization; Epigenetic modulators; Targeting the exosome pathway	IGF2BP2 inhibitors; C3aR1 antagonists; Histone modification inhibitors; DNA methylation modulators; Bisphosphonates (e.g., Denosumab)

## Data Availability

No new data were created or analyzed in this study.

## Abbreviations

A2AR	Adenosine A2A Receptor
ADRB1	Adrenoceptor Beta 1
apCAFs	Antigen-Presenting Cancer-Associated Fibroblasts
APC	Antigen-Presenting Cell
APOC2	Apolipoprotein C2
APOE	Apolipoprotein E
BLCA	Bladder Cancer
CAFs	Cancer-Associated Fibroblasts
CAR T	Chimeric Antigen Receptor T-cell
CCL18	C-C Motif Chemokine Ligand 18
CCL2	C-C Motif Chemokine Ligand 2
CCR1	C-C Motif Chemokine Receptor 1
CCR2	C-C Motif Chemokine Receptor 2
CD	Cluster of Differentiation
CD163	Cluster of Differentiation 163
CD206	Cluster of Differentiation 206
CD44	Cluster of Differentiation 44
CD68	Cluster of Differentiation 68
CEBPB	CCAAT/Enhancer Binding Protein Beta
Circ-0034880	Circular RNA 0034880
C1QC	Complement C1q C Chain
CRC	Colorectal Cancer
CSF1	Colony-Stimulating Factor 1
CSF1R	Colony-Stimulating Factor 1 Receptor
CSCs	Cancer Stem Cells
CXCL	C-X-C Motif Chemokine Ligand
CXCL10	C-X-C Motif Chemokine Ligand 10
CXCL12	C-X-C Motif Chemokine Ligand 12
CXCL8	C-X-C Motif Chemokine Ligand 8
CXCR	C-X-C Motif Chemokine Receptor
DCs	Dendritic Cells
ECM	Extracellular Matrix
ecmCAFs	Extracellular Matrix-associated Cancer-Associated Fibroblasts
EIRS	Epigenetic Immune-Related Scoring
EMT	Epithelial–Mesenchymal Transition
EVs	Extracellular Vesicles
FAP	Fibroblast Activation Protein
FABP1	Fatty Acid Binding Protein 1
FN1	Fibronectin 1
GBM	Glioblastoma
GEO	Gene Expression Omnibus
GPNMB	Glycoprotein NMB
GREM1	Gremlin 1
H3K18	Histone 3 Lysine 18
H3K4me3	Histone 3 Lysine 4 Trimethylation
HCC	Hepatocellular Carcinoma
HIF-1α	Hypoxia-Inducible Factor 1-alpha
HNSCC	Head and Neck Squamous Cell Carcinoma
HPSCC	Hypopharyngeal Squamous Cell Carcinoma
IFN-γ	Interferon Gamma
IGF2BP2	Insulin-like Growth Factor 2 mRNA-Binding Protein 2
iCAFs	Proinflammatory Cancer-Associated Fibroblasts
IL-12	Interleukin 12
IL-1β	Interleukin 1 Beta
IL-1	Interleukin 1
IL-4I1	Interleukin 4 Induced 1
IL-6	Interleukin 6
INHBA	Inhibin Beta A
IRF5	Interferon Regulatory Factor 5
ITGA5	Integrin Subunit Alpha 5
ITGAV	Integrin Subunit Alpha V
ITGB1	Integrin Subunit Beta 1
LRRC15	Leucine Rich Repeat Containing 15
LRP1	Low-Density Lipoprotein Receptor-Related Protein 1
MAFB	MAF BZIP Transcription Factor B
MAIT	Mucosal-Associated Invariant T
MDMs	Monocyte-Derived Macrophages
MDP	Monocyte and Dendritic Cell Progenitor
MDSCs	Myeloid-Derived Suppressor Cells
MHC-II	Major Histocompatibility Complex Class II
MIF	Macrophage Migration Inhibitory Factor
MMP2	Matrix Metalloproteinase 2
MMP9	Matrix Metalloproteinase 9
m6A	N6-methyladenosine
mTOR	Mechanistic Target of Rapamycin
MYBL2	MYB Proto-Oncogene Like 2
myCAFs	Myofibroblast-like Cancer-Associated Fibroblasts
NAMPT	Nicotinamide Phosphoribosyltransferase
NF-κB	Nuclear Factor Kappa B
NSCLC	Non-Small Cell Lung Cancer
PD-1	Programmed Cell Death Protein 1
PDAC	Pancreatic Ductal Adenocarcinoma
PI3K	Phosphatidylinositol-4,5-Bisphosphate 3-Kinase
PLAU	Plasminogen Activator, Urokinase
PLIN2	Perilipin 2
PLXDC1	Plexin Domain Containing 1
PMN	Pre-Metastatic Niche
PitNETs	Pituitary Neuroendocrine Tumors
POSTN	Periostin
PRAME	Preferentially Expressed Antigen in Melanoma
PTGER4	Prostaglandin E Receptor 4
scRNA-seq	Single-Cell RNA Sequencing
SPIC	Spi-C Transcription Factor
SPP1	Secreted Phosphoprotein 1
STAT1	Signal Transducer and Activator of Transcription 1
STAT3	Signal Transducer and Activator of Transcription 3
TAMs	Tumor-Associated Macrophages
TCGA	The Cancer Genome Atlas
TEVs	Tumor-Derived Extracellular Vesicles
TGF-β1	Transforming Growth Factor Beta 1
TGF-βR1	Transforming Growth Factor Beta Receptor 1
THC	Tetrahydrocurcumin
TIB	Tumor Immune Barrier
TME	Tumor Microenvironment
TNBC	Triple-Negative Breast Cancer
TPSCs	Tumor-Associated Pancreatic Stellate Cells
TREM2	Triggering Receptor Expressed on Myeloid Cells 2
TP53	Tumor Protein P53
VEGF	Vascular Endothelial Growth Factor
VEGFA	Vascular Endothelial Growth Factor A
VSIG4	V-Set Immunoglobulin Domain Containing 4
WDR5	WD Repeat Domain 5
ZCCHC12	Zinc Finger CCHC-Type Containing 12
